# Draft Genome Sequences of *Bacillus cereus* E41 and *Bacillus anthracis* F34 Isolated from Algerian Salt Lakes

**DOI:** 10.1128/genomeA.00383-17

**Published:** 2017-05-18

**Authors:** Mohamed Seghir Daas, Albert Remus R. Rosana, Jeella Z. Acedo, Farida Nateche, Salima Kebbouche-Gana, John C. Vederas, Rebecca J. Case

**Affiliations:** a Valcore Laboratory, Department of Biology, University M’Hamed Bougara of Boumerdès, Boumerdès, Algeria; b Food Technology Research Division, Institut National de la Recherche Agronomiqued' Algérie, El Harrach, Algiers, Algeria; c Department of Biological Sciences, University of Alberta, Edmonton, Alberta, Canada; d Department of Chemistry, University of Alberta, Edmonton, Alberta, Canada; e Microbiology Group, Laboratory of Cellular and Molecular Biology, Faculty of Biological Sciences, USTHB, Bab Ezzouar, Algiers, Algeria

## Abstract

Two strains of *Bacillus*, *B. cereus* E41 and *B. anthracis* F34, were isolated from a salt lake in Aïn M’lila-Oum El Bouaghi, eastern Algeria, and Ain Baida-Ouargla, southern Algeria, respectively. Their genomes display genes for the production of several bioactive secondary metabolites, including polyhydroxyalkanoate, iron siderophores, lipopeptides, and bacteriocins.

## GENOME ANNOUNCEMENT

*Bacillus* is a large and ubiquitous genus commonly isolated from soil and sediment habitats. *Bacillus* spp. are aerobic or facultative anaerobic Gram-positive rod-shaped bacteria capable of forming spores, which allows them to persist and disperse in diverse habitats. *Bacillus* spp. also produce a variety of bioactive small molecules, and we report here the genome sequences of two isolates, which encode the production of polyhydroxyalkanoate, iron siderophores, lipopeptides, and bacteriocins.

Genomic DNA was extracted from single-colony isolates using the DNeasy blood and tissue kit (Qiagen), according to the manufacturer’s protocol. Sequencing libraries from the genomic DNA extracts were prepared using the Nextera XT DNA library preparation kit (Illumina). Whole-genome sequencing was performed using the MiSeq reagent kit version 2 (2 × 250 cycles) and MiSeq sequencing technology (Illumina), generating 150-bp paired-end reads. *De novo* assembly of the reads into contiguous sequences (contigs) was done using the CLC Genomics Workbench version 7.5.2 (CLC bio, Aarhus, Denmark). All of the genomes sequenced exceeded 300× coverage. The draft genomes of E41 and F34 yielded 29 and 66 contigs, respectively. The draft genomes were then annotated using RAST version 2.0 (1) or PGAP (http://www.ncbi.nlm.nih.gov/genome/annotation_prok). Species identities were determined by an average nucleotide identity (ANI) of >97% using JSpecies version 1.2.1 (2) with previously sequenced genomes in the GenBank database. Secondary metabolites were predicted using antiSMASH version 4 (3). The potential to produce bacteriocins was detected using BAGEL3 (4).

Genome annotation by the NCBI PGAP predicted 5,779 genes, including 5,492 coding sequences (CDSs), 12 rRNAs, and 98 tRNAs, in the genome of *Bacillus cereus* E41, and 6,265 genes, including 5,866 CDSs, 10 rRNAs, and 76 tRNAs, in the genome of *Bacillus anthracis* F34.

Both *Bacillus* spp. have the complete gene cluster for polyhydroxyalkanoate biosynthesis that is used for the production of bioplastics. They are also predicted to produce the siderophore petrobactin (*asbABCDEF*) (5). In addition, they both contain the bacillibactin nonribosomal peptide synthetase (NRPS) operon *dhb*ACEF and the corresponding iron-bacillibactin uptake cluster *feuABCD*-*yuiI* (6).

The* B. cereus* E41 genome contains a complete gene cluster for the lantibiotic thusin (*thsA1TM1A2A2′M2FE*) (7). Furthermore, the genome contains biosynthetic gene clusters for surfactin, polyoxypeptin, as well as sugar compounds, such as S-glycan and exopolysaccharides.

### Accession number(s).

The whole-genome shotgun projects have been deposited in DDBJ/ENA/GenBank for *B. cereus* E41 (MTAT00000000) and *B. anthracis* F34 (MTAU00000000).
